# The Presence of Flour Affects the Efficacy of Aerosolized Insecticides used to Treat the Red Flour Beetle, *Tribolium castaneum*

**DOI:** 10.1673/031.010.19601

**Published:** 2010-11-10

**Authors:** Michael D. Toews, James F. Campbell, Franklin H. Arthur

**Affiliations:** ^1^ USDA-ARS Grain Marketing and Production Research Center, 1515 College Ave, Manhattan, KS, 66502–2736 USA; ^2^ Current Address: University of Georgia, Department of Entomology, 122 S. Entomology Dr., Tifton, GA, 31793–0748 USA

**Keywords:** fogging, integrated pest management, methyl bromide replacement

## Abstract

Experiments were conducted in tightly sealed pilot scale warehouses to assess the efficacy of common aerosolized insecticides on all life stages of *Tribolium castaneum* (Herbst) (Coleoptera: Tenebrionidae) when exposed in dishes containing 0 to 2 g of wheat flour either under pallets or out in the open. Petri dishes containing 0, 0.1, 1, or 2 g of flour were prepared with 25 eggs, 3^rd^ instars, pupae, or adults and then immediately treated with aerosolized solvent, Pyrethrins, or esfenvalerate. Twenty-four h after insecticide exposure, the dishes were brought to the laboratory and placed in a growth chamber and held for a 3 day moribund (knockdown) assessment and a 21 day mortality assessment. Mortality in untreated controls was generally less than 10%, with the exception of the 21 day counts of adults and eggs. Solvent-treated replications followed similar trends, except that additional mortality was observed in exposed larvae and pupae. In the insecticide-treated dishes, mortality of *T. castaneum* provisioned with flour generally showed a linear decrease with increasing flour deposits. Regardless of life stage, mortality did not exceed 60% when individuals were exposed in petri dishes containing 2 g of flour. Exposure location also made a significant difference in observed mortality. While mortality never exceeded 75% in dishes positioned under pallets, there was never less than 80% mortality in dishes exposed in the open. Although there was a perceptible increase in mortality with esfenvalerate compared to Pyrethrins, these differences were considerably less than the variation observed among flour deposits. The study suggests that sanitation and preparation prior to aerosol insecticide treatments were more important than choice of a particular insecticide.

## Introduction

Aerosol insecticide applications, also called fogging, can be an important component of stored-product pest management programs for food facilities such as mills, food warehouses, and processing plants. This application method involves dispensing a liquid insecticide formulation through a mechanical device to atomize the particles so that they are dispensed as a fog or mist, usually with particle sizes in the range of 5 to 50 μ?m (Peckman and Arthur 2005). Application of the aerosolized insecticide can be conducted using handheld fog generators, temporary floor standing units, or wall mounted permanent systems. Under label guidance, pest management professionals do not remove bulk ingredients and finished products from facilities during these applications. Aerosols are most commonly used as components of integrated pest management (IPM) programs. In addition, aerosols are used as a fumigation alternative when the facility cannot be made gas tight or production schedules do not permit an extended shut down. Historically, pest management professionals utilized aerosolized applications of dichlorvos, but resistance to this insecticide has been reported (Arthur et al. 1988, Halliday et al. 1988). Use of reduced risk aerosol insecticides such as Pyrethrins, synthetic pyrethroids, and insect growth regulators is increasing in the food industry, but limited information is available on their efficacy.

There are a number of recent reports describing dispersion and efficacy of aerosols in small-scale studies and on artificially introduced pest populations in commercial facilities (Arthur 2008; Arthur and Campbell 2008; Jenson et al. 2009). However, with the exception of Toews et al. (2006), there are little or no data in the peer reviewed scientific literature regarding impact of aerosol insecticides on resident pest populations of food facilities. Interpretation of insect captures obtained through trapping studies in food facilities is confounded by importation of untreated products, insect immigration through openings in structure such as shipping doors, and cleaning routines. Changes in conditions over time and physical differences among commercial facilities make replication difficult. These factors make it challenging to accurately evaluate efficacy of different aerosol insecticides based on trapping data.

The red flour beetle, *Tribolium castaneum* (Herbst) (Coleoptera: Tenebrionidae), is one of two major economic insect pests in cereal processing, warehouse, and retail facilities (the other being Indianmeal moth, *Plodia interpunctella*). Flour and other products from processed cereals are the preferred diets of *T. castaneum* (Arbogast 1991). In previous studies, applications of non-aerosolized residual insecticides in a controlled environment failed to eliminate resident *T. castaneum* populations, though they did produce increased mortality and reduced adult emergence depending on the specific insecticide (Toews et al. 2005b, 2009). In this study, the efficacy of aerosol insecticides against different life stages of *T. castaneum* were directly assessed using the same controlled environment (tightly sealed pilot scale warehouses), but exposed the insects in confined petri dishes that could be removed after treatment. The specific objectives of this study were to determine differences in efficacy between two specific aerosol insecticides against different life stages of *T. castaneum*, and to examine the effect of food accumulation and level of coverage on efficacy.

## Materials and Methods

All insects were exposed in washed 9 cm (diameter) plastic petri dishes prepared with small quantities of flour to simulate spilled food accumulation. Previously frozen white bread flour was added to dishes in quantities of 0, 0.1, 1.0, or 2.0 g each. The 0.1 g flour deposits were created by placing a few grams into a petri dish and then lightly inverting the dish to discard all but the flour that adhered to the plastic surface; this amount was confirmed using a microbalance. The larger flour deposits were created by weighing the flour and then pouring it into a pile in the center of the dish. Immediately prior to treatment with insecticides, 25 *T. castaneum* eggs, larvae (3^rd^ instars), pupae, or adults (<2 wk old) were added to each individual petri dish (one dish for each flour accumulation by life stage treatment for a total of 16 dishes). During each aerosol application, a complete set of petri dishes was located in the center of the room while a second complete set of dishes was positioned 1 m away under a wooden pallet (1219 by 1016 mm) covered by a corrugated cardboard pallet slip sheet and empty cardboard boxes stacked to a height of 600 mm above the top of the pallet. Therefore, there were a total of 32 uncovered petri dishes in each warehouse, and each dish contained 25 test insects of a given life stage.

The *T. castaneum* insect colony used for the study was founded with ∼?100 immatures from a Midwestern US food production facility; the insect colony was maintained for four years in the laboratory at 27.0 ±±0.5 °° C and 65 ±± 5% RH on wheat flour fortified with 5% brewer's yeast (ICN Biomedicals, icnbiomed.com).

### Fogging application

Replicated aerosol applications were applied using a professional fogger in sealed pilot scale warehouses. The pilot scale warehouses (32.9 to 40.5 cubic meters each) were previously described in Toews et al. (2005). Three of the warehouses measured 5.9 m long by 2.8 m wide by 2.0 meters tall, while the fourth warehouse was 5.9 m long by 2.8 m wide by 2.4 meters tall. Interior surfaces including the ceiling of the warehouses were lined with overlapping pieces of 0.15 mm thick polyethylene sheeting to prevent insecticide buildup on the physical structure. Aerosol applications were made using a model E-2 (Whitmire Micro-Gen Research Laboratories, Inc., www.pestcontrol.basf.us/) dispersal unit that produced droplets in the range of 1–30 microns. The quantity of product in the fogger reservoir was weighed before and after application to ensure the calculated dosage was administered. Chemical treatments included an untreated negative control, a positive control composed of the aerosolized insecticide carrier solvent (Isopar™™ M Fluid, Exxon Mobil Chemical Co., www.exxonmobilchemical.com.) and two aerosolized insecticides. The insecticides consisted of 1% Pyrethrins, 2% piperonyl butoxide, 2.94% N-octyl bicycloheptene dicarboximide (Prescription Treatment®® brand ULD®® BP-100, Whitmire Micro-Gen Research Laboratories), and 3.48% esfenvalerate (Conquer Residual Insecticide Concentrate, Paragon Professional Pest Control Products, paragonprofessional.com). Insecticides were applied at the maximum label rate and dosage (29.6 ml undiluted ULD®® BP-100 per 28.3 cubic meters and 29.6 ml of 0.027% (AI) Conquer per 28.3 cubic meters). Conquer was diluted in Isopar®® M Fluid (hereafter referred to as solvent) but ULD®® BP-100 concentrate was sold ready for use. Application of the proper amount of insecticide solution required approximately 70 sec per warehouse. Between experimental applications, a minimum of 30 ml (∼?1 min run time) of clean solvent was dispensed into an empty warehouse to clean residual insecticide out of the fogging unit. The assignment of insecticide to warehouse was randomized with each application, and there were a total of four replications of each insecticide treatment combination.

### Mortality assessment

Following insecticide application, pilot-scale warehouses remained sealed for 24 h to allow the insecticide droplets to settle. Following the settling period, petri dishes covered with lids were brought to the laboratory where they were placed in an environmental chamber maintained at 28°° C and 60% RH with a 14:8 L:D photoperiod. Dishes that did not receive any flour prior to insecticide application received 1.0 g of clean flour to sustain live insects. Individual adults and larvae in each dish were scored as live or moribund (knocked down or not capable of walking) at 3 days post application. Dishes containing eggs or pupae during insecticide exposure were not examined at 3 days post application because it was difficult to discern any movement or color change by these immobile life stages. Regardless of life stage, all individuals were assessed for mortality at 21 days post application. For adults and larvae, percent recovery was calculated by subtracting the proportion of dead individuals at 21 days from the proportion moribund at 3 days post application. Environmental conditions inside the untreated warehouse were monitored continuously using a data logger (Hobo H8, Onset Computer, www.onsetcomp.com).

### Statistical analyses

Data were analyzed independently by insect life stage as a split plot design, with insecticide as the main plot and quantity of flour and location under a pallet or out in the open as the subplot treatments. For analysis purposes, numbers of dead insects were expressed as a proportion of total insects exposed. With one exception, the arcsine square root transformation was employed for all response variables prior to analyses to normalize variances (Zar 1999). The transformation was not used on the percent recovery response variable because those values were negative in some cases, and therefore could not be transformed. Although both an untreated and positive control (solvent only) was included with each replicate, mortality in these two treatments was expected to be negligible; therefore, these data were statistically compared with each other, but not included in the statistical comparisons with insecticide treatments because the direct comparisons between insecticide treatments and the remaining experimental variables were focused on. Additionally, low mortality in the untreated vs. high mortality in the insecticide-treated replication would result in obvious interactions with other experimental factors (i.e. flour deposits by insecticide or exposure location by insecticide) that may not be relevant. Statistical comparisons among insecticide treated replicates were conducted using a generalized linear models approach (Proc Mixed) (SAS 9.1 software, SAS Institute), with date and date by insecticide modeled as random variables. Degree of freedom adjustments followed the methods of Kenward and Roger (1997). Effects of flour deposition in petri dishes were further characterized using linear and quadratic trend analyses. Coefficients for these orthogonal contrasts with unequally spaced data were generated using PROC IML (SAS 9.1 software). Comparisons between untreated and solvent-treated treatments were accomplished using analyses of variance with date and date by insecticide modeled as random variables. Differences among treatments were considered significant at áá = 0.05. Regardless of transformation used during data analyses, untransformed means (converted to a percent) and standard errors were presented in the text and on all figures.

## Results

A mean of 40.0 ±± 4.0 ml of insecticide solution in the BP-100 replicates, 38.7 ±± 9.8 ml in the Conquer replicates, and 38.8 ±± 4.5 ml in the solvent-only replicates (*n* = 4 per insecticide) were applied. By volume, this was 5 to 10% more material than the target dose, but some of that material was expected to have remained inside the aerosol dispenser and aspirator hose that extended into the insecticide reservoir. Therefore, it was concluded that all warehouses received the full labeled dose of the insecticides on a volume of space basis. Environmental conditions at the time of insecticide application ranged from 19 to 24°° C and during the 24 h post-application period ranged from 19-35°° C (42-63% RH) across all applications.

Percentage of moribund (3 day) and dead (21 day) individuals in untreated controls was generally low (<10%) with two exceptions: adults and eggs at 21 day post-exposure had 19.4 and 23.8% mortality, respectively (Table 1). Replications treated with solvent only yielded a low percentage moribund and dead, except there was significant increase (6-fold) in the observed mortality of larvae and pupae in the 21 day counts compared to the 3 day counts (Table 1).

**Table 1.**
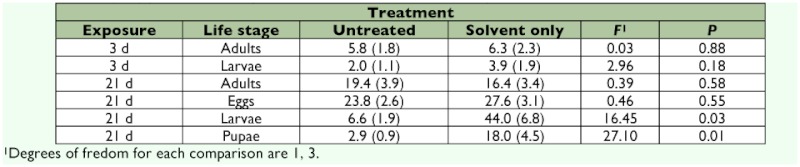
Mean (+SE) percent mortality in untreated and solvent treated replications.

**Figure 1.**
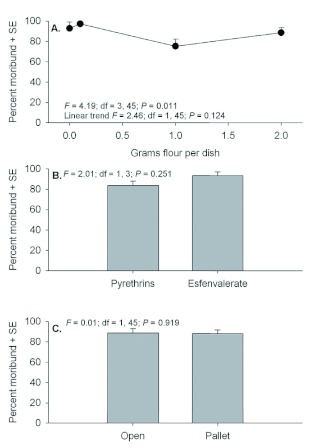
Percent moribund (+SE) *Tribolium castaneum* adults assessed 3 d post application when exposed in petri dishes containing 0, 0.1, 1.0, or 2.0 g flour deposits (A), with Pyrethrins vs. esfenvalerate (B), and exposure location in the open or under a pallet (C). High quality figures are available online.

**Figure 2.**
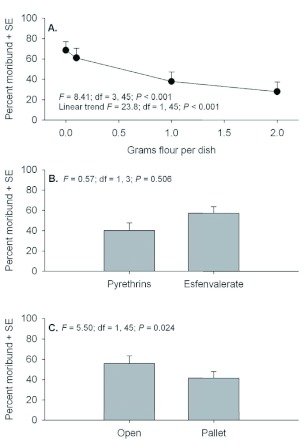
Percent moribund (+SE) *Tribolium castaneum* larvae assessed 3 d post application when exposed in petri dishes containing 0, 0.1, 1.0, or 2.0 g flour deposits (A), with Pyrethrins vs. esfenvalerate (B), and exposure location in the open or under a pallet (C). High quality figures are available online.

Although 3 day post-exposure moribund counts were conducted for mobile life stages, these data should be interpreted cautiously because of differences with the 21 days postexposure data. Examination of moribund counts of adult *T. castaneum* 3 days after exposure showed no evidence of significant interactions (*F* = 0.00 to 1.44; *P* = 0.586 to 0.244). Significant main effects were detected for quantity of flour in petri dishes, but there were no differences between insecticides or exposure locations (Figure 1). Despite a significant effect for flour, there was no evidence of a linear trend in mortality relative to increasing flour amounts. Similar to adult counts, moribund counts of larvae 3 days after exposure showed no evidence of interactions (*F* = 0.05 to 0.79; *P* = 0.503 to 0.432), but significant effects were detected among flour deposits and between exposure locations (Figure 2). Although not statistically compared, moribund larvae counts appear to be considerably less than moribund adult counts.

**Figure 3.**
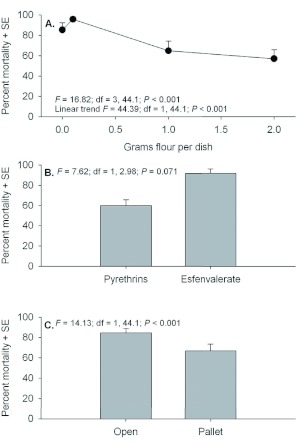
Percent mortality (+SE) of *Tribolium castaneum* adults assessed 21 d post application when exposed in petri dishes containing 0, 0.1, 1.0, or 2.0 g flour deposits (A), with Pyrethrins vs. esfenvalerate (B), and exposure location in the open or under a pallet (C). High quality figures are available online.

Mortality at 21 days post-exposure is a good indicator of treatment efficacy and there were similar responses to individuals exposed as adults and larvae. Statistics pertaining to the mortality of adults showed no interactions (*F* = 0.90 to 2.43; *P* = 0.359 to 0.071). However, significant main effects were detected among flour deposits and between exposure locations (Figure 3). A strong linear trend showing decreasing mortality with increasing amounts of flour deposits was evident. Mortality in esfenvalerate-treated dishes averaged greater than 90% and mortality in pyrethrins-treated dishes averaged 60%, but this difference was marginally non-significant (*P* = 0.071). There was an 18% decrease in mortality of adults exposed under pallets compared to individuals exposed out in the open. There were also no interactions among factors in the 21 day mortality counts of individuals exposed as larvae (*F* = 0.43 to 0.59; *P* = 0.514 to 0.628).

**Figure 4.**
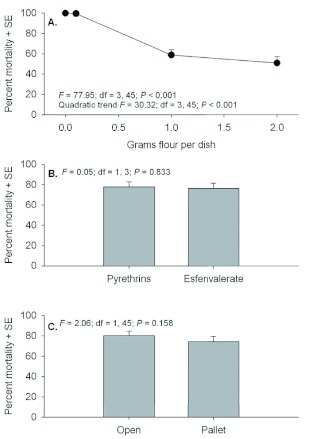
Percent mortality (+SE) of *Tribolium castaneum* larvae assessed 21 d post application when exposed in petri dishes containing 0, 0.1, 1.0, or 2.0 g flour deposits (A), with Pyrethrins vs. esfenvalerate (B), and exposure location in the open or under a pallet (C). High quality figures are available online.

In this case, significant effects were detected among flour deposits and between exposure locations (Figure 4). Similar to adults, there was a strong linear trend showing decreasing mortality with increasing flour deposits, and the difference in mortality between Pyrethrins and esfenvalerate was marginally nonsignificant (*P* = 0.058).

**Figure 5.**
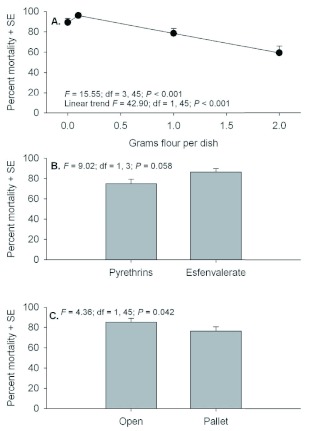
Percent mortality (+SE) of *Tribolium castaneum* eggs assessed 21 d post application when exposed in petri dishes containing 0, 0.1, 1.0, or 2.0 g flour deposits (A), with Pyrethrins vs. esfenvalerate (B), and exposure location in the open or under a pallet (C). High quality figures are available online.

**Figure 6.**
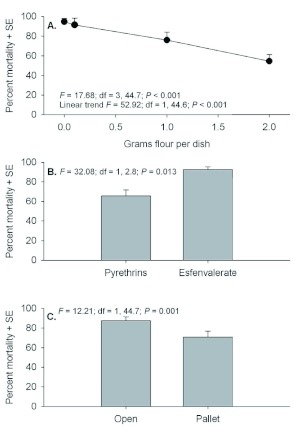
Percent mortality (+SE) of *Tribolium castaneum* pupae assessed 21 d post application when exposed in petri dishes containing 0, 0.1, 1.0, or 2.0 g flour deposits (A), with Pyrethrins vs. esfenvalerate (B), and exposure location in the open or under a pallet (C). High quality figures are available online.

All treatment effects were also examined for individuals exposed as eggs and pupae. For individuals exposed as eggs there were no interactions (*F* = 0.02 to 1.22; *P* = 0.312 to 0.882). Significant main effects were detected among flour deposits, with a quadratic trend evident (Figure 5); the quadratic trend showed that the mortality rate decreased at a faster rate between 0 and 1 g flour deposits than between 1 and 2 g deposits. Mortality of individuals exposed as eggs was virtually identical between insecticide treatments and between the two exposure locations. For individuals exposed as pupae there were no interactions among factors in the 21 day mortality counts of (*F* = 0.60 to 2.09; *P* = 0.620 to 0.115). Significant differences were detected among flour deposits, between insecticides, and between exposure locations (Figure 6). Mortality in dishes containing 2 g flour deposits was about 40% less than observed mortality when no flour was present in the dishes. A 25% increase in mortality was observed for individuals exposed to esfenvalerate compared with Pyrethrins, while across insecticide treatments there was a 15% increase in mortality to individuals exposed in the open compared with exposure under a pallet.

**Figure 7.**
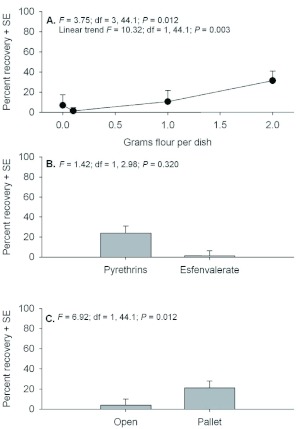
Percent recovery (+SE) of *Tribolium castaneum* adults between 3 and 21 d post application when exposed in petri dishes containing 0, 0.1, 1.0, or 2.0 g flour deposits (A), with Pyrethrins vs. esfenvalerate (B), and exposure location in the open or under a pallet (C). High quality figures are available online.

There were no interactions among main effects for recovery of adults (*F* = 0.73 to 1.26; *P* = 0.540 to 0.298). Significant main effects were detected for differences among flour deposits and between exposure locations (Figure 7). Recovery generally increased linearly with increasing flour deposits with a range of 30% between 0.1 g and 2 g deposits. Recovery of individuals exposed under pallets increased by more than 15% compared with individuals exposed in the open. There were no significant interactions or significant main effects when testing for differences in the recovery of larvae (*F* = 0.06 to 2.46; *P* = 0.075 to 0.761).

## Discussion

The overall trend across the full range of flour deposits was for decreased mortality with increasing flour deposits. However, *T. castaneum* mortality actually increased slightly when comparing percentage mortality of adults and larvae in dishes containing 0 and 0.1 g flour deposits. This phenomenon occurred with adults at both 3 and 21 days post exposure and larvae at 21 days post exposure (Figures 1A, 3A, and 4A). The authors hypothesize that this is simply an artifact of the test conditions. Individuals that were exposed in petri dishes without flour deposits during insecticide exposure had to receive some flour or they would starve before the 21 day mortality assessments; therefore, 1.0 g of clean flour was added 24 h after exposure. However, the literature shows that addition of clean food will enable adult *T. castaneum* to survive after they have been exposed to an insecticide (Arthur 2000a, b).

Here, examination of the percent recovery of adults (Figure 7A) shows that recovery of adults exposed with no flour exceeded the recovery of adults with 0.1 g flour deposits. The impact of food is important because immature life stages will typically be located in refugia patches in a warehouse or processing plant. The difficulty in predicting mortality of a particular insecticide application is confounded by the fact residual food cannot generally be cleaned out of all floor cracks and hidden refugia. The greater the proportion of the population in a warehouse or processing plant that is not directly exposed to the insecticide, i.e. in hidden refugia or in contact with food during or after exposure, the less likely aerosol treatments are going to be effective. Sanitation is therefore a critically important step in pest management interventions.

Mortality of *T. castaneum* individuals in dishes positioned under pallets generally decreased compared to those exposed in the open. Since aerosol droplets tend to settle downward and horizontal obstructions such as equipment, pallets, and shelving can inhibit the deposition of the insecticide. However, horizontal drift of these very small particles could produce mortality even when the test subjects are hidden under structures (Jenson et al. 2009). The presence of processing equipment and products blocking aerosol deposition is the most plausible reason for observations in field studies where adults were always captured immediately following aerosolized insecticide applications (Toews et al. 2006). Efficacy should increase following good sanitation and in empty warehouses. The authors suspect that actual insecticide dose reaching the test insects in these tightly sealed warehouses likely exceeded the dose that would reach insects in a typical field situation.

In a direct comparison of two commonly used aerosolized insecticides, no differences in mortality were observed at 3 days, but esfenvalerate was slightly more efficacious in the 21 day assessments. The fact that the *P-*values were strongly significant in one case and marginally non-significant in two more, suggests that the synthetic pyrethroid insecticide could have some additional activity compared to the synergized Pyrethrins. Under usual test conditions *P-*values between 0.05 and 0.1 would not merit attention. However, this trend may be real based on the lack of statistical power that occurs when there are only 3 degrees of freedom in the denominator as occurred in the main plot treatments of this split plot. Increasing degrees of freedom (i.e. increasing replication) will increase the ability to detect statistical differences. Nevertheless, comparison of the mean range of percent mortality (21 day counts) attributed to flour deposition (41%) was more than double the mean range attributed to insecticide type (20%).

In the untreated and solvent treated replications, mortality generally varied between two and 20 percent with several exceptions. In 21 day post-exposure counts of adults from untreated replications 20% mortality may seem relatively high, but three weeks is a fairly long time for the entire test population to survive in close proximity to each other, which creates some extenuating circumstances. Pati and Yan (2003) showed that confined *T. castaneum* adult females rapidly mated with multiple males within a short period of time, which produced a proportionate decrease in life span with number of copulation events. We expect that surviving individuals in the untreated and oil treated replicates mated many more times than the few surviving individuals in the insecticide treated replicates. Mortality of untreated eggs is likely a completely separate issue. While 25 eggs were counted, handled, and introduced into each petri dish, each egg may not have been viable. Arbogast (1991) reported that about 90% of *T. castaneum* eggs are viable at optimal conditions. Also, *T. castaneum* larvae are cannibalistic and will feed on eggs of their own species (Sokoloff 1974) thereby reducing the population. In this test, crowding effects were likely not important sources of mortality in the insecticide-treated replications because the insecticides greatly decreased the number of individuals and relative crowding. Mortality in solvent-treated replications suggested that the solvent may cause some moderate mortality of larvae and pupae. Eggs notwithstanding, one hypothesis is that the deposition of an oily film on immature life stages may have clogged the spiracles and led to asphyxiation. The surface tension of the hydrocarbon based solvent is only 0.02–0.025 N/m at 20°° C compared with water's surface tension of 0.073 N/m (Avallone and Baumeister III 2009). Hence, solvent is a much better ““wetting agent”” and will therefore fill very small pores, like body openings, rather than bridging them with surface tension.

Data shown here strongly suggest that level of sanitation before insecticide application was more important than use of a particular insecticide. Excellent sanitation also forces insects to move around (Roesli et al. 2003; Toews et al. 2005a) and could thereby increase the potential for insecticide contact. Campbell and Hagstrum (2002) found that *T. castaneum* spend much more time in food patches than outside food patches. Pest managers could not only reduce cost, but may also increase the efficacy of insecticide treatments if they emphasize preparation and sanitation of storage facilities prior to aerosol applications.
